# 

**Published:** 1838-01-01

**Authors:** 


### CONTENTS

OF THE

### MEDICO-CHIRURGICAL REVIEW,

### No. LV. JANUARY 1, 1838,

[BEING No. XV. OF A DECENNIAL SERIES.]

### REVIEWS.

I.

### DISEASES OF THE RECTUM.

1. A Treatise on the Malformations, Injuries, and Diseases of the Rectum and Anus. By GEORGE BUSHE, M.D.

2. On the Diseases of the Rectum. By JAMES SYME, F.R.S.E. .. 1

1. Malformations of the Rectum and Anus .. 3

A. Imperforation of the Anus .. 4

B. Imperforation of the Rectum .. 4

C. Unnatural Terminations of the Rectum .. 4

D. Termination of other Organs in the Rectum .. 5

E. Absence of the Rectum .. 5

F. TREATMENT .. 6

2. Foreign Bodies in the Rectum .. 9

3. Laceration of the Rectum .. 13

A. Incomplete Laceration .. 13

B. Complete Laceration .. 14

4. Inflammation of the Rectum .. 18

5. Inflammation and Excoriation of the Rectum .. 20

6. Inflammation of the Rectum and Anus, arising from the Application of Gonorrhœal Matter .. 20

7. Fissure of the Anus .. 21

A. Treatment .. 23

8. Neuralgia of the Extremity of the Anus .. 24

9. Spasmodic Contraction of the Sphincter Ani .. 26

A. Simple Spasmodic Contraction .. 26

B. Spasmodic Contraction, with Affection of the Genito-urinary Organs .. 29

10. Ulceration of the Rectum .. 31

II.

### DISEASES OF THE CHEST.

1. A Treatise on the Diagnosis and Treatment of Diseases of the Chest. By WILLIAM STOKES, M.D. .. 33

1. Bronchitis .. 35

A. Acute Primary Bronchitis .. 37

B. Chronic Primary Bronchitis .. 38

C. Physical Signs of Bronchitis .. 41

D. Acute Secondary Bronchitis .. 44

E. Chronic Secondary Bronchitis .. 45

F. Treatment of Bronchitis .. 47

### III.

### DISEASES OF THE WINDPIPE.

1. Observations on the Surgical Pathology of the Larynx and Trachea, including Remarks on Croup, Cynanche Laryngea, &c. By Mr. W. H. Porter .. ..

2. A Treatise on the Diseases and Injuries of the Larynx & Trachea. By Mr. F. Ryland

3. Traité Pratique de la Pthisie Laryngée, &c. Par MM. Trousseau et Belloc .. } 50

   1. Croup .. .. .. .. . .. .. .. .. .. .. .. .. .. .. .. 50

   2. Dipthérite .. .. .. .. .. .. .. .. .. .. .. .. .. .. .. 5[illegible]

   3. Cynanche Maligna .. .. .. .. .. .. .. .. .. .. .. .. .. 5[illegible]

   4. Phthisis Laryngea .. .. .. .. .. .. .. .. .. .. .. .. . 6[illegible]

### IV.

### ON THE TREATMENT OF THE VENEREAL DISEASE BY MERCURY.

1. Practical Observations on the Venereal Disease and on the Use of Mercury. By A. Colles, M.D. .. .. .. .. .. .. .. .. .. .. .. .. .. .. ..

2. The Works of John Hunter, F.R.S., with Notes. Edited by Mr. J. F. Palmer.. } 6[illegible]

   1. On the Administration of Mercury .. .. .. .. .. .. .. .. .. .. 6[illegible]

   2. On the Methods of administering Mercury .. .. .. .. .. .. .. 7[illegible]

### V.

Dr. Blake on Monomania .. .. .. .. .. .. .. .. .. .. .. .. .. .. .. .. 8[illegible]

### VI.

An Exposition of the Signs and Symptoms of Pregnancy, the Period of Human Gestation, and the Signs of Delivery. By Dr. Montgomery .. . .. .. .. .. .. 90

   1. Effects of Pregnancy .. .. .. .. .. .. .. .. .. .. .. .. .. .. .. 91

   2. Signs and Symptoms of Pregnancy .. .. .. .. .. .. .. .. .. .. .. 96

      A. Presumptive Signs of Pregnancy .. .. .. .. .. .. .. .. .. 9[illegible]

      B. Probable Signs of Pregnancy .. .. .. .. .. .. .. .. .. .. .. 100

      C. Unequivocal Signs of Pregnancy .. .. .. .. .. .. .. .. .. 10[illegible]

### VII.

On the Action of Diuretic Medicines. By Dr. C. G. Mitcherlich .. .. .. .. .. 10[illegible]

   1. Diuretics whose Physiological Action is to produce an increased Secretion of Urine.. .. .. .. .. .. .. .. .. .. .. .. .. .. .. .. .. 110

   2. Diuretics whose Therapeutic Action occasions an increased Secretion of Urine 113

### VIII.

### RECENT PUBLICATIONS ON ANATOMY.

I. Observations on the Structure and Functions of the Spinal Cord. By R. D. Grainger, Esq. .. .. .. .. .. .. .. .. .. .. .. .. .. .. .. ..

II. & III. Guy's Hospital Reports, Nos. 4 and 5 .. .. .. .. .. .. .. ..

IV. Elements of Physiology. By J. Muller, M.D. Translated from the German, by William Baly, Esq. Part I. .. .. .. .. .. .. .. .. .. .. ..

V. Elements of Anatomy. By Jones Quain, M.D. Part I... .. .. .. .. ..

VI. Anatomical Plates. By Jones Quain, M.D. .. .. .. .. .. .. .. .. } 11[illegible]

   1. On the Structure and Functions of the Spinal Cord .. .. .. .. .. .. 11[illegible]

   2. Properties of the Grey and Fibrous Substances .. .. .. .. .. .. .. 121

   3. Anatomy of the Spinal Cord and Nerves .. .. .. .. .. .. .. .. .. 123

   4. Physiology of the Spinal Cord .. .. .. .. .. .. .. .. .. .. .. .. 130

      A. Of the Vertebral Portion of the Spinal Cord .. .. .. .. .. .. .. 131

      B. Of the Cranial Portion of the Spinal Cord .. .. .. .. .. .. .. 132

IX.

Surgical Observations on Tumors, with Cases and Operations. By J. C. Warren, M.D. 136

1. Tumors of the Glands.. .. .. .. .. .. .. .. .. .. .. .. 136

A. Tumors of Lymphatic Glands .. .. .. .. .. .. .. .. .. .. 137

B. Tumors of the Secreting Glands .. .. .. .. .. .. .. .. .. 142

### PERISCOPE.

### Bibliographical Notices.

1. An Essay on Pyrexia, or Symptomatic Fever, as illustrative of the Nature of Fever in General. By H. Clutterbuck, M.D. .. .. .. .. .. .. .. .. .. .. 153

Treatment of Pyrexia in general .. .. .. .. .. .. .. .. .. .. .. 158

2. Philosophy of Living. By Mr. Mayo and Dr. Ticknor .. .. .. .. .. .. .. 161

3. Medical Science and Ethics: an Introductory Lecture delivered at the Bristol Medical School. By W. O. Porter, M.D. .. .. .. .. .. .. .. .. .. .. 163

4. Treatment of Cholera .. .. .. .. .. .. .. .. .. .. .. .. .. .. .. 164

5. Changes produced in the Nervous System by Civilization, &c. By Robert Verity, M.D. .. .. .. .. .. .. .. .. .. .. .. .. .. .. .. .. 166

6. A Treatise on the Influenza of 1837, as observed at Birmingham. By P. Blakiston, M.A. .. .. .. .. .. .. .. .. .. .. .. .. .. .. .. 168

1. State of the different Organs .. .. .. .. .. .. .. .. .. .. .. .. 169

7. The Flora of Jamaica; or a Description of the Plants of that Island. By J. Macfadyen, M.D... .. .. .. .. .. .. .. .. .. .. .. .. .. .. .. 170

8. Remarks on the Treatment of Fractures of the Lower Extremities without the Aid of Splints. By J. F. Burke, Esq. .. .. .. .. .. .. .. .. .. .. .. 171

9. Elements of Chemistry. By the late Edward Turner, M.D. Sixth Edition .. .. 176

### Clinical Review, and Hospital Reports.

KENT AND CANTERBURY HOSPITAL.

1. Report of Medical Cases treated at the Kent and Canterbury Hospital during the Year 1836-7. By John M'Divitt, M.D. .. .. .. .. .. .. .. .. .. .. 177

1. On Fevers .. .. .. .. .. .. .. .. .. .. .. .. .. .. .. .. 186

A. Simple continued Fever .. .. .. .. .. .. .. .. .. .. .. .. 190

B. Typhoid Fever .. .. .. .. .. .. .. .. .. .. .. .. .. .. 191

C. Chlorotic Fever .. .. .. .. .. .. .. .. .. .. .. .. .. .. 192

D. Fever from Prolonged Lactation .. .. .. .. .. .. .. .. .. 205

2. Dumfries and Galloway Royal Infirmary .. .. .. .. .. .. .. .. .. 205

3. Gleanings during Three Years spent in the Indian Seas. By Mr. W. B. Marshall, 209

A. Swan River and King George's Sound .. .. .. .. .. .. .. .. .. 209

B. Norfolk Island .. .. .. .. .. .. .. .. .. .. .. .. .. .. .. 212

GUY'S HOSPITAL.

4. Guy's Hospital Reports. No. 5.. .. .. .. .. .. .. .. .. .. .. .. 213

A. Cases of Gangrene, Aneurism, &c. By Mr. Bransby Cooper .. .. .. 215

ST. GEORGE'S HOSPITAL.

5. Division of the Tendo-Achillis. Is it a new operation? .. .. .. .. .. .. .. 221

6. Treatment of Varicose Veins of the Leg .. .. .. .. .. .. .. .. .. .. 221

7. Tables and Calculations relating to Rheumatism. By Dr. M'Leod .. 223
A. Liability of different Ages .. 223
B. Duration of Complaint .. 223
C. Occurrence of Pericarditis .. 223

### Spirit of the Foreign Periodicals, &c.

1. Illustrations of Phrenology .. 225

A. Prefatory Remarks .. 225

B. Caractères Phrenologiques .. 228

2. Character of Cuvier .. 236

3. Notice of the New French Phrenological Journal.—M. Broussais on Education, &c. 238

4. Phrenology does not sanction a Belief in Fatalism .. 241

5. Alleged Affection of the Organs of Calculation .. 243

6. Apoplexy—Loss of the Memory of Words .. 243

7. Delirium—Alleged Affection of certain Organs .. 244

8. Demonomania, with Delirium .. 245

9. Account of Vito Mangiamele, the youthful Prodigy of Calculation—the phrenological Development of his Head, &c. .. 247

10. Organization of the Brain in the Negro compared with that in the European, and also in the Ourang Outang. By Professor Tiedemann .. 249

A. Weight of the Brain in the European .. 249

B. The Brain of the Negro .. 251

11. On Encephalic Irritation, Arachnitis, Hydrocephalus Acutus, &c. in Infants. By M. Piorry .. 252

12. M. Louis on the Value of Auscultation as a Means of Diagnosis .. 257

13. M. Bricheteau on Phthisis Pulmonalis .. 265

14. Influence of Climate on Phthisis Pulmonalis .. 267

15. Case of Acute Phthisis—Analysis of the Cases reported in M. Louis' Work—Reflections on the Subject. By Dr. D'Espine .. 270

A. Duration .. 273

B. Pathological Anatomy .. 273

C. Symptoms .. 274

16. Commentaries on some of the Aphorisms of Hippocrates. By M. Guerbois .. 276

### Miscellanies.

1. Surgical Report of some Cases in the County of Clare Infirmary. By George O'Brien, M.D. .. 281

A. Case of Retention of Urine from Contraction of the Orifice of the Urethra, 281

B. Compound Fracture of the Elbow .. 281

C. Fatal Injury of the Leg .. 282

D. Gun-shot Wound of the Popliteal Artery .. 283

E. Wound of the Pharynx .. 283

F. Poisoning by Sulphuric Acid .. 284

2. Dr. Dickson and Dr. Johnson .. 285

Bibliographical Record .. 286

### Appendix.

On a peculiar and undescribed Injury of the Shoulder, being the Substance of a Clinical Lecture delivered December 2, by Mr. Guthrie .. 289

